# Specific Extracellular Vesicles, Generated and Operating at Synapses, Contribute to Neuronal Effects and Signaling

**DOI:** 10.3390/ijms25105103

**Published:** 2024-05-07

**Authors:** Jacopo Meldolesi

**Affiliations:** 1 IRCCS San Raffaele Hospital, Vita-Salute San Raffaele University, 20129 Milan, Italy; meldolesi.jacopo@hsr.it; 2 CNR Institute of Neuroscience, Milano-Bicocca University, 20854 Vedano al Lambro, Italy

**Keywords:** cargo, canonical, clathrin, dendrite, Drosophila, endocytosis/endosomes, exocytosis, navigation, pre-/post-synaptic, spine, subtype

## Abstract

In all cell types, small EVs, very abundant extracellular vesicles, are generated and accumulated within MVB endocytic cisternae. Upon MVB fusion and exocytosis with the plasma membrane, the EVs are released to the extracellular space. In the central nervous system, the release of neuronal EVs was believed to occur only from the surface of the body and dendrites. About 15 years ago, MVB cisternae and EVs were shown to exist and function at synaptic boutons, the terminals’ pre- and post-synaptic structures essential for canonical neurotransmitter release. Recent studies have revealed that synaptic EVs are peculiar in many respects and heterogeneous with respect to other neuronal EVs. The distribution of synaptic EVs and the effect of their specific molecules are found at critical sites of their distribution. The role of synaptic EVs could consist of the modulation of canonical neurotransmitter release or a distinct, non-canonical form of neurotransmission. Additional roles of synaptic EVs are still not completely known. In the future, additional investigations will clarify the role of synaptic EVs in pathology, concerning, for example, circuits, trans-synaptic transmission, diagnosis and the therapy of diseases.

## 1. Introduction

In the central nervous system (CNS), extracellular vesicles (EVs) operate primarily as message carriers across boundaries, inducing versatile interactions among neuron and glial cell types, relevant in physiological and pathological processes. In the various brain areas, the frequency and impact of EVs are variable [1,2]. For example, EVs of neuronal origin are abundant in the medulla, cerebellum and occipital regions [2]; EVs from microglia are low in most brain areas, except for in the hippocampus and post-central gyrus; EVs from astrocytes, present in all areas, are abundant in both the occipital gyrus and medulla [2].

In addition to their peculiar functions, EVs from the two types of glial cells participate in many neuronal functions [1]. EVs from microglia participate in the screening of neuronal parenchyma, searching for alterations in its homeostasis and ensuing implications in neurodegenerative diseases [3,4]; astrocyte EVs participate in most neuronal functions, including synaptic development and activity [5,6]. Other neuronal functions are regulated/activated by the combined activity of EVs from astroglia and microglia [7,8]. Many other brain processes are governed by EVs released only by neurons from different brain areas, with some illustrating biological activities, including neuronal secretion, and others being involved in pathological processes [9,10,11].

The present Introduction Section has summarized some general properties of brain cells and their EVs. The subsequent section, Section 2, summarizes the mechanisms of the EV membrane and cargo release from neurons, including their generation, release and navigation up to the binding of their targets. The properties of synaptic EVs are illustrated in Section 3. Their emerging functions, reported in terms of key mechanisms and critical results, accounting for almost 50% of the text, are shown in Section 4.

## 2. Generation and Segregation of EVs Are Followed by Their Release to the Extracellular Space

The initial site of EV generation is assembled by endocytosis, a process activated on the cell surface, operating in many critical functions. Plasma membrane internalization, dependent/independent of critical proteins such as clathrin and caveolin, results in the generation of early endosomal cisternae of variable size and surface specificity. Among the cell processes activated in these cisternae are the retrieval of synaptic vesicles upon neurotransmitter secretion, the uptake of nutrients, the activation of surface receptors and many others.

In terms of EV development, the major contributions of endocytosis include its direct participation and the assembly of molecular components with the ensuing luminal segregation. Enlarged endocytic cisternae, as well as established interactions with their molecular exchanges, are matured by the luminal budding of small vesicles delimited by specific membranes, rich in endosomal proteins, such as annexins, flotillin and the tetraspannin CD63, together with bioactive lipids. In the cargoes, the plethora of signaling molecules include various membrane-associated proteins, together with soluble proteins, both ESCRT-dependent and -independent [10,11]. Other proteins include growth and transcription factors and several types of lipids, together with coding and non-coding RNAs, including various forms of mRNAs and miRNAs, active from their luminal location [12,13,14].

For some time after their assembly, MVBs undergo traffic within the cytoplasm of the cells of their origin. The process that follows, stimulated by cell activation, includes the transfer of MVBs close to the cell surface, followed by the fusion of their membrane with the plasma membrane, with the ensuing exocytosis followed by the release of segregated EVs to the extracellular space [15]. Upon their release, the EVs start navigating in the extracellular medium, reaching, within the target cells of their specific binding, sites spread at variable distances. Most often, such binding is established with neurons distinct from those of their last EV generation. Thus, the specific EV interactions result in the transient establishment, on the surface or upon internalization, of inter-neuronal interactions, with the ensuing activation of specific functions [13,15,16].

## 3. Release of EVs to the Extracellular Space

As already specified in Section 2, MVB fusion with the plasma membrane occurs by a peculiar form of exocytosis, followed by the spontaneous release of pre-segregated EVs from the MVB lumen to the extracellular space. Here, the presentation is primarily focused on the synapses. At least some of the MVB exocytoses occur as a result of the use of tools analogous to those involved in neurotransmitter release, including the machinery of SNARE/synaptobrevin proteins and tethering factors, which are also active in many cancer cells [17,18]. The Rab11 GTPase has been shown to participate in at least some forms of CNS cells, as well as in governing their extracellular EV traffic [19]. Another Rab form, Rab 27a, has been shown to control dendritic spine formation at the post-synaptic sites of the Barrel cortex [20].

The initial discovery of MVB and EVs, made in Drosophila [21,22], demonstrated their localization at synapses, in particular in the synaptic boutons, structures well known for their other function, neurotransmitter release. Because of the discovery of EVs, synaptic boutons are now known for two analogous functions, the neurotransmitter release and the EV release processes, both governed by Ca2+ dynamics but inducing different responses [23]. The key differences come from the luminal materials of the two small vesicles and their distinct lives. In particular, EVs, upon their release from MVBs, survive during navigation in the extracellular space, finally reaching and interacting with their cell targets. The properties of the latter processes are discussed in the next section.

## 4. The Unique Role of Synaptic EVs

**Figure 1:**
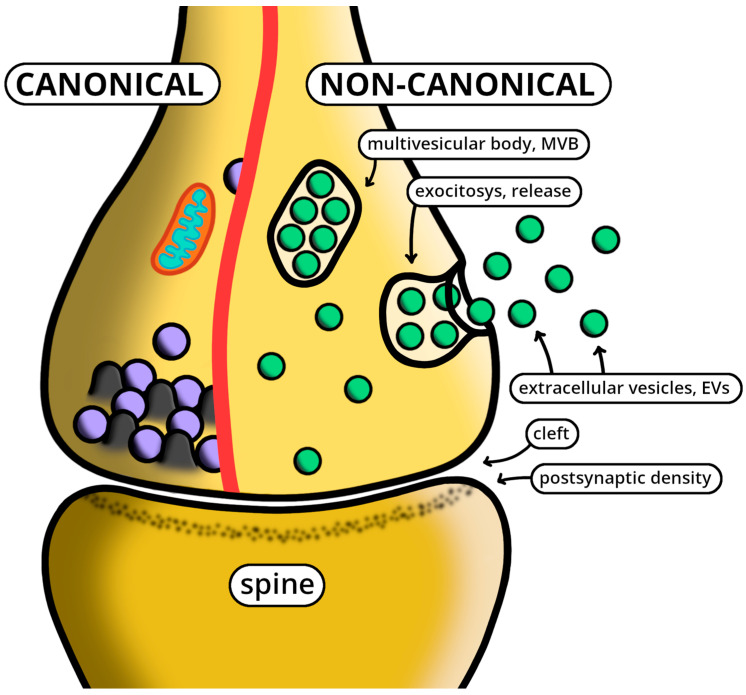
Separate presentation, in a synapse, of the two forms: the canonical form, with the release of neurotransmitters, and the non-canonical form, showing the accumulation and release of EVs.

As already emphasized in Section 3, neuronal EV release, previously known as a single form spread to the large surface of cells, is now recognized to include another process, specifically located at synapses. The recognition of the new processes at synaptic boutons [21,22,23] led to the revealing of the local specificity of its synaptic EVs, analogous in some properties but distinct in many others with respect to the canonical endocytic processes of neurotransmitter release and membrane recycling (Figure 1) [24]. Analogous distinctions have been demonstrated in investigations of protein and DNA/RNA binding pathways during the development of synapses. Specifically, the coexistence of canonical and non-canonical EV processes has been found to change during the development of dendritic spines [25].

In the pre-synaptic terminal (top), the two forms are shown separate from each other by an arbitrary red line. The canonical section (left) includes neurotransmitter vesicles released at active zones. The non-canonical section (right) shows the MVB, an endocytic cisterna filled with many EVs. Upon MVB fusion and exocytosis with the plasma membrane, the EVs are released to the extracellular space. The free EVs, accumulated in the cytoplasm, are due to the uptake of navigating vesicles into synapses (not shown). The thin space between the pre- and post-synaptic structures is the cleft, possibly including materials from both synaptic forms. The post-synaptic terminal is shown as a single spine, in direct continuity with a dendritic fiber (not shown here), believed to receive signals and materials from both pre-synaptic forms.

The progressive information established about the endocytic development of synaptic EVs has reinforced the interpretation of their signaling. In particular, EVs derived from neurons have been confirmed to exhibit components such as synaptic proteins, lipids and RNAs. Their induced generation of dendritic spines depends on relevant signals such as those induced by TrkB, with trophic effects in the spotlight for synaptic plasticity modulation [26,27]. Such inter-neuronal signaling is able to connect pre- and post-synaptic neuronal sites by the diffusion of small EVs, i.e., by the variety of signaling molecules contained in the EV vesicle cargoes. Signaling between neurons is more complex than previously believed. It includes not only the specific chemical signals of distinct conventional synapses but also “a packet” of active cargo molecules released by EVs [28]. The problems discussed during the last two years include the possible interactions established between the two types of transmission [28]. From a functional point of view, the role of EVs with their targets has been conceived in at least two general terms: rapid modulators of classical synaptic activity and slow regulators of neuronal plasticity. A promising interpretation favors EVs as novel regulators of neurotransmitter function (Figure 1) [29].

So far, the events presented in synaptic environments have been focused primarily on specific EV processes. In the following sites, the presentation is not limited to EVs but rather extended to the classical synapses and their strategy. The properties of the latter processes are well known. Following the exocytosis of neurotransmitter vesicles, concentrated at active zones, their membranes and associated proteins need to be recycled. The endocytic machinery of such a process is distributed in the periactive zone (PAZ), adjacent to the active zones of exocytosis. The processes of various PAZ regions have been found to correspond to distinct functions, suggesting a correlation between various PAZ subdomains and sites of ongoing processes. These data suggest the neuronal exocytic and endocytic recycling activities to be spatially correlated [30]. Similar conclusions were reached by an analysis of a specific protein, the activity-related cytoskeletal protein (Arc), associated with EVs in pre-synaptic terminals. Interestingly, Arc was found to be present, at different levels however, in both the pre-synaptic boutons and post-synaptic spines, i.e., at the two terminal structures of synapses. Within both structures, the site of Arc concentration was found to correspond to either the synaptic cleft or sites close to synapses, supporting the possible direct transport of the protein from one dynamic site to another [31].

A final interpretation has been proposed about the participation of neural EVs operating downstream of BDNF, brain-derived neurotrophic factor [32]. The latter factor has been found to mediate the expression of three miRNAs, miR-132-5p, miR-128-5p and niR-690, which, in turn, increase excitatory synapse assembly. Concomitantly, BDNF increases the expression of additional EVs together with synaptic vesicle clustering, thereby increasing synaptic transmission together with synchronous EV neuronal activity. In other words, an integrated process induces significant increases in both synaptic transmission and synchronous EV neuronal activity [32]. Finally, initial interest has been shown regarding the possible role of synaptic EVs in various diseases (neurodegenerative diseases and neuropsychiatric disorders) [19,27,32,33], with effects from diagnoses to relevant therapies. Detailed EV studies, however, have mostly reported about whole brain cells [33,34], not yet about synaptic vesicles. Synaptic EV studies focused on diseases therefore remain to be carried out.

## 5. Conclusions

The present, comprehensive short review illustrates the neuronal EVs generated and active at pre-synaptic terminals. These neuronal EVs, identified about 15 years ago [21,22], have been investigated from 2021 [18,19,20,23,24,25] up to now [26,27,28,29,30,31,32]. The EVs generated at synapses appear different from those generated in other neuronal areas. Therefore, at single neurons, synaptic EVs appear heterogeneous, as previously reported for other types of cells [35,36,37].

In functional terms, the role of synaptic EVs appears highly relevant. Their activities emerge from a communication platform, active in signaling organization [30]. Their observed operation could be a modulation of canonical neurotransmission signaling. Alternatively, their contribution could be independent due to a non-canonical form of neurotransmission [29]. For either type of contribution, EV activity could participate in circuit connectivity, dynamically operating downstream of BDNF [32].

The various functions of EVs could be relevant for synapses due to their possible involvement in wireless communications, protein trans-synaptic transmission, structural organization and other processes [28,29,30,31]. In the future, studies in these fields might be expanded. The same could occur for studies about the diagnosis and therapy of diseases, already considered here, however only at the pre-synaptic level [33,34]. As reported in Section 1 [5,6], in most brain cases, neuronal EVs operate together with glial EVs, especially with astroglial EVs. Although, to the best of my knowledge, possibilities of this type have never been reported in the literature for synaptic EVs; nevertheless, they remain of potential interest, deserving attention in the future.

## Abbreviations

Arc: activity-related cytoskeletal protein; BDNF, brain-derived neurotrophic factor; CNS, central nervous system; EV, extracellular vesicle; ILV, intra-luminal vesicle; MVB, multi-vesicular body; PAZ, periactive zone.
